# Supplementary material for: TNF stimulation primarily modulates transcriptional burst size of NF‐κB‐regulated genes
Source: Mol Syst Biol. 2021 Jul 20;17(7):e10127. doi: 10.15252/msb.202010127 (PMC8290835; doi:10.15252/msb.202010127)
Supplement: Supplementary file 1 — Appendix [file MSB-17-e10127-s002.pdf]

Appendix: TNF stimulation primarily modulates transcriptional burst size of NF-κB-regulated genes

Appendix Figure S1: smFISH images of NF-κB target genes before and after TNF stimulation and image filtering…………………………………………………………………………...2

Appendix Figure S2: Appendix Figure S2. Validation of parameters used to fit the two-state model……………………………………………………………………………………………4

Appendix Figure S3: Comparing pooled bootstrapping to replicate averages of smFISH data…..5

Appendix Figure S4: Stochastic run with two independent promoter sites with bursty transcription……………………………………………………………………………………6

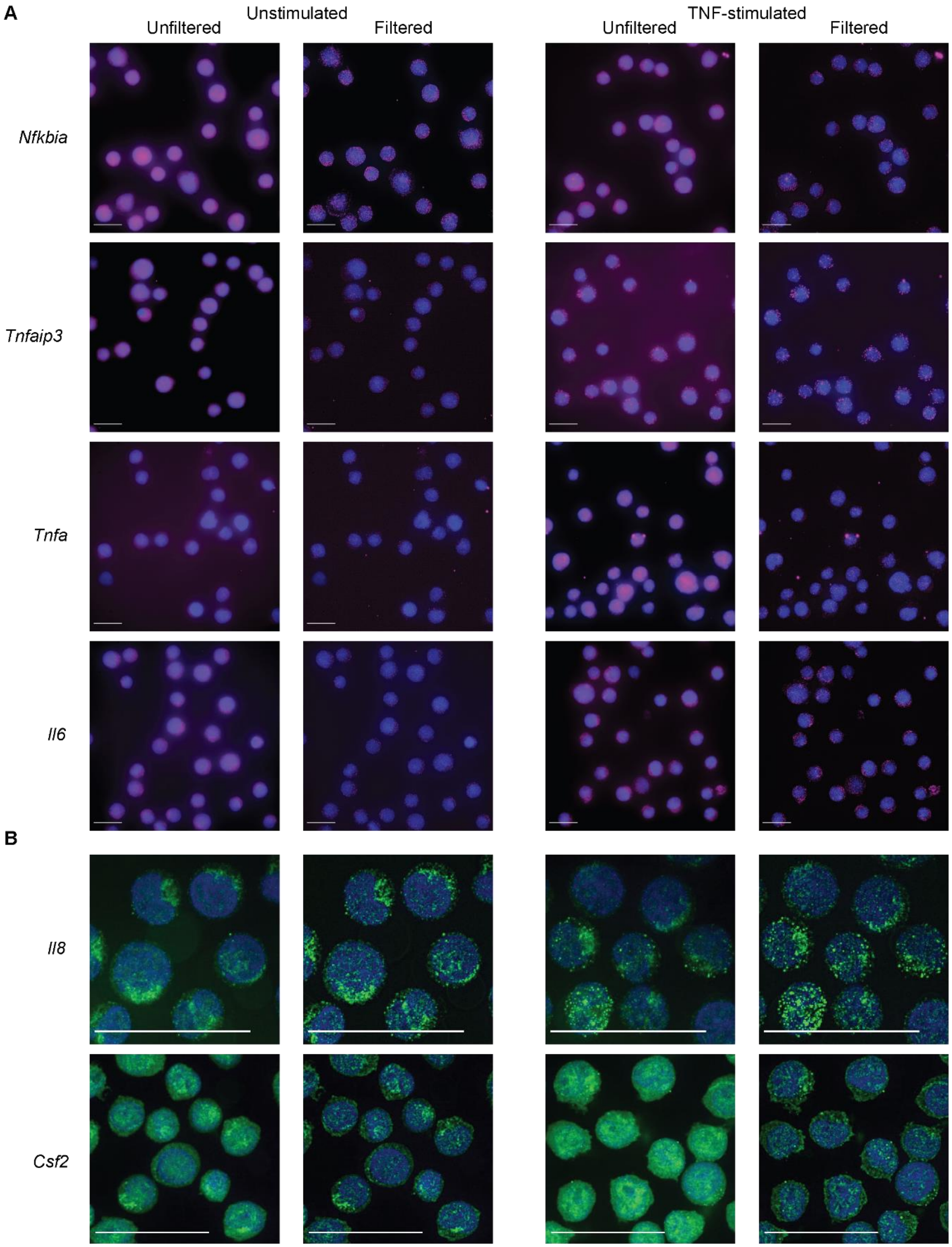

Appendix Figure S1. smFISH images of NF-κB target genes before and after TNF stimulation and image filtering.

A-B Maximum intensity projections of z-stacks of cells stained for the indicated genes before (left two columns) or after (right two columns) stimulation with 20 ng/mL TNF. Images were captured on either a widefield (A) or spinning disk confocal (B) microscope. Spinning disk images were captured using regions of interest of variable size to minimize the amount of blank space. Images are displayed before and after Dual Gaussian filtering in FISH-Quant. Brightness and contrast were enhanced for visualization. Scale bars: 20 μm.

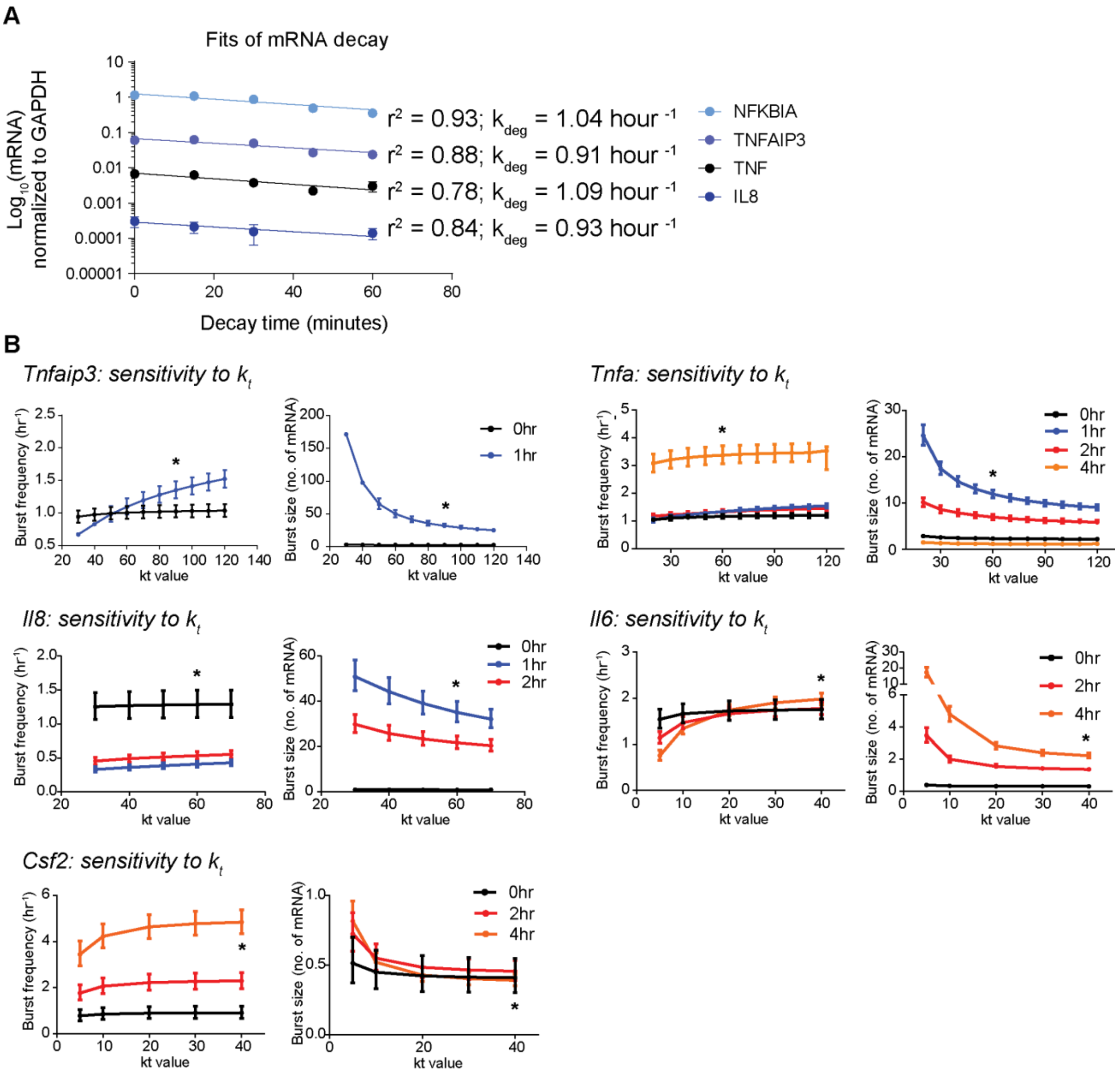

Appendix Figure S2. Validation of parameters used to fit the two-state model.

A Jurkat T cells were stimulated with 20 ng/mL TNF for 1 hour followed by treatment with 10 µg/mL Actinomycin-D for 0, 15, 30, 45, or 60 minutes and mRNA levels for the indicated target genes were measured by RT-qPCR. Exponential mRNA decay rates were fit using non-linear regression. Data are presented as mean ± SD of three biological replicates. Goodness of fit (r^2) and decay rate constant (kdeg, units of 1/hour) are displayed.
B Sensitivity analysis of how fitted burst frequency (left) and burst size (right) values vary with the value of k_t used in the model. Error bars indicate bootstrapped 95% CIs. The value of k_t used for each gene is marked with an asterisk.

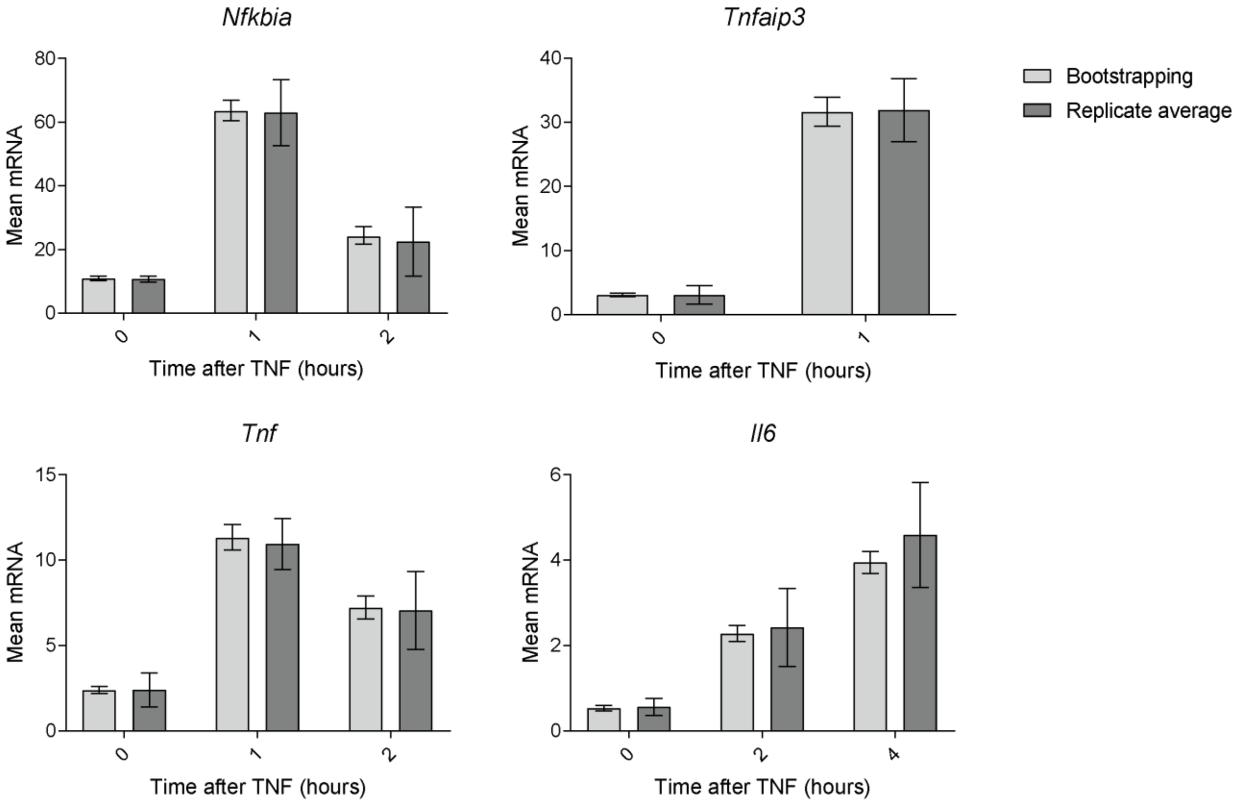

Appendix Figure S3. Comparing pooled bootstrapping to replicate averages of smFISH data.

Comparisons of mean expression from smFISH data when either pooling all replicate experiments for bootstrapping or when calculating means for each replicate experiment and then calculating the mean and standard deviation. Data are presented as mean +/- %95 confidence interval for bootstrapping (light grey) or as mean +/- standard deviation for replicate averages (dark grey). Three replicate smFISH experiments each with n > 100 cells are included for all genes and timepoints.

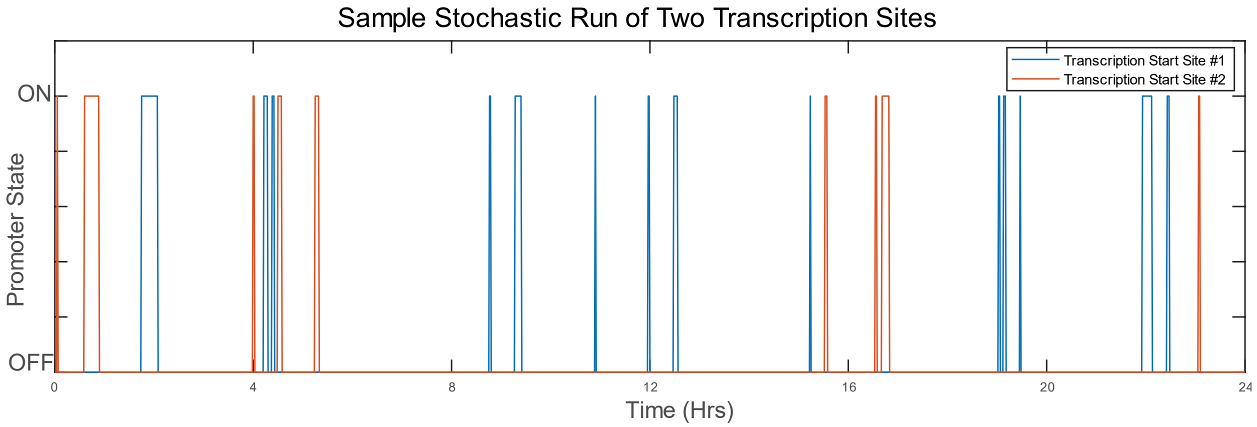

Appendix Figure S4. Stochastic run with two independent promoter sites with bursty transcription.

Temporal comparisons of two transcription sites stochastically simulated with NFSIM for 24 hours. For this simulation only, the burst size followed original assumptions as described in the Materials and Methods, whereas the burst frequency was halved to maintain similar transcript numbers.
