# Supplementary material for: TNF stimulation primarily modulates transcriptional burst size of NF‐κB‐regulated genes
Source: Mol Syst Biol. 2021 Jul 20;17(7):e10127. doi: 10.15252/msb.202010127 (PMC8290835; doi:10.15252/msb.202010127)
Supplement: Supplementary file 2 — Expanded View Figures PDF [file MSB-17-e10127-s004.pdf]

Expanded View Figures

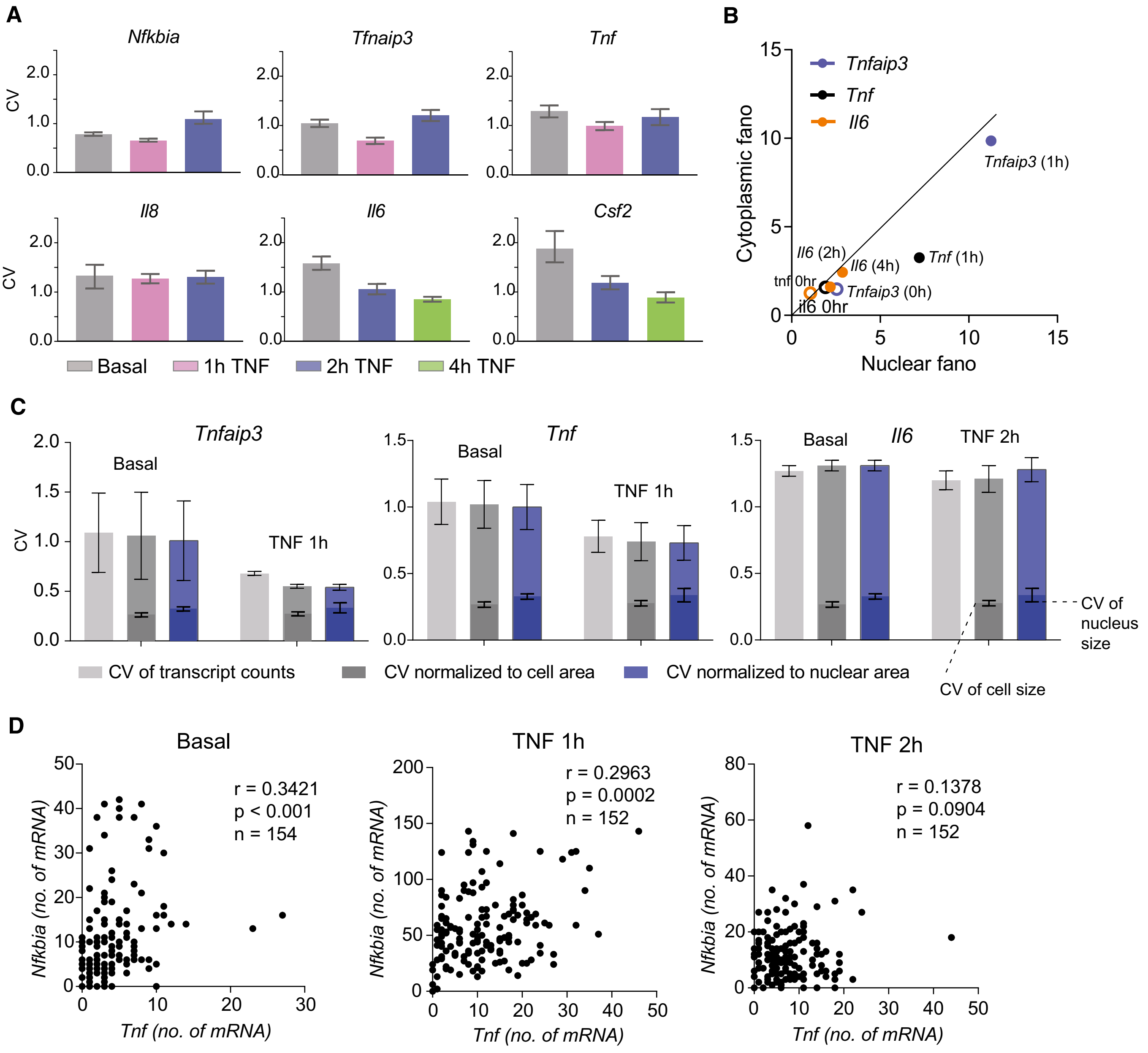

Figure EV1. TNF treatment induces differential changes in noise across target genes that is not explained by cellular sources of variation (related to Fig 2).

A Bar graphs of coefficient of variation (CV) of basal and TNF-stimulated smFISH distributions presented in Fig 2B and C for the indicated genes at the indicated time points. Error bars indicate bootstrapped 95% confidence intervals (CIs). Significant differences indicated by non-overlapping CIs. Sample sizes are displayed in Fig 1E and 2C.

B Fano factor of mRNA counts in the nuclear vs cytoplasmic fractions of cells before and after 20 ng/ml TNF stimulation for the indicated genes. Data points are labeled with gene and time point. The diagonal is shown as a solid line to guide the eye.

C CV of basal and TNF-stimulated smFISH distributions for *Tnfaip3*, *Tnf*, and *Il6* presented in Fig 2B and C unnormalized (light gray) or normalized to cellular area (dark gray) or nuclear area (blue). Lower bars indicating the CV of cellular area (dark gray) and nuclear area (blue) in basal and TNF-stimulated smFISH images are included with each gene for reference. Data are presented as mean ± SD of CVs from two replicate experiments for each gene and condition.

D Scatter plots comparing mRNA counts for *Tnf* and *Nfkbia* in the same cells from multiplex smFISH for basal, 1 h and 2 h of 20 ng/ml TNF stimulation. Pearson correlation *r*, *P*-value, and no. of cells *n* are indicated.

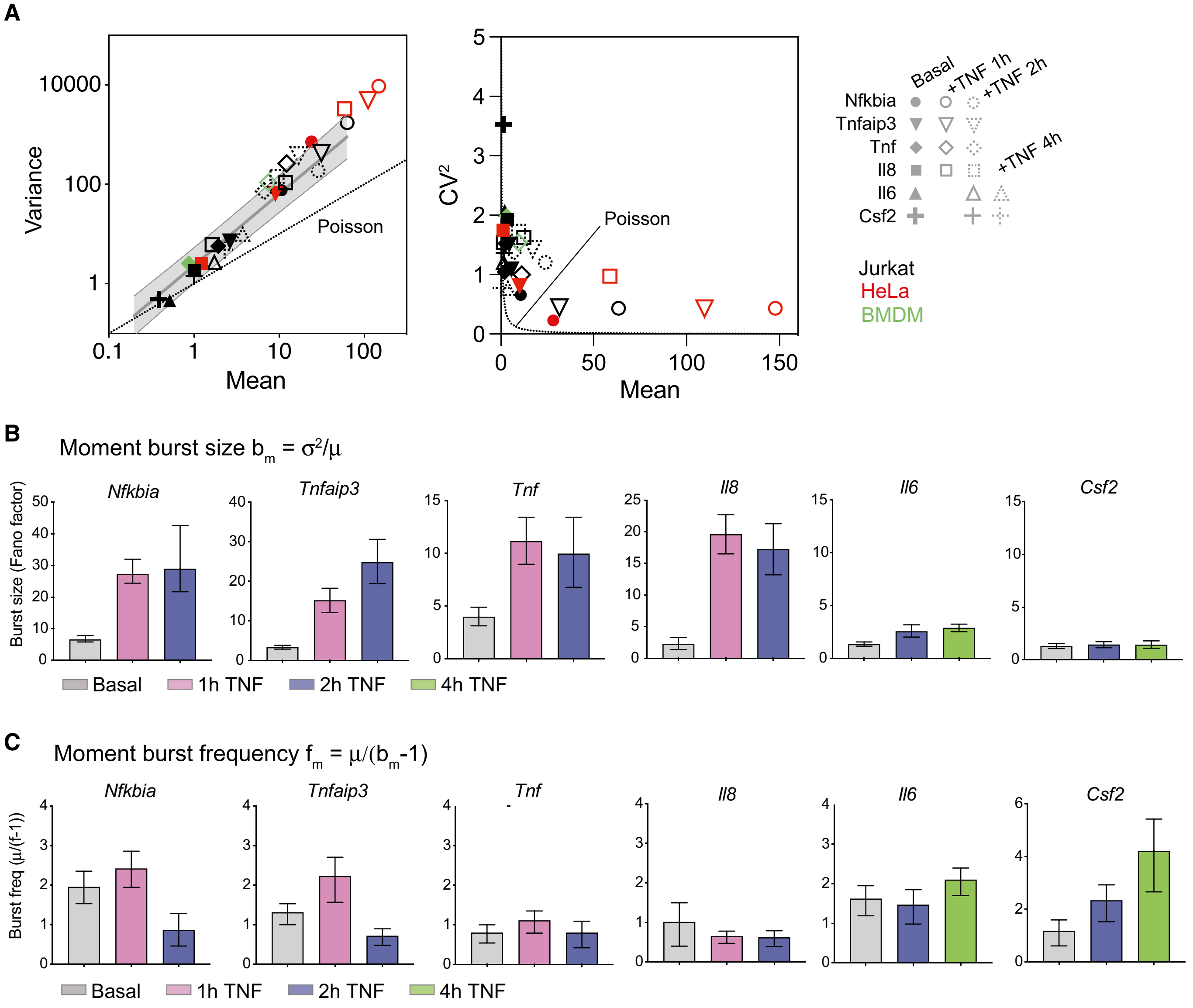

Figure EV2. Target genes exhibit mean-variance and mean-CV^2 trends and moments distinct from Poisson behavior and consistent with transcriptional bursting (related to Fig 3).

A Graph of variance vs mean (left) and CV^2 vs mean (right) for endogenous gene targets before (black) and after treatment with 20 ng/ml TNF in Jurkat T cells, HeLa cells, or murine bone marrow–derived macrophages (see labels for details). Gray shading indicates 95% CI of basal trend line. Poisson trend line indicated by dashed lines. HeLa data from Lee *et al* (2014).

B, C Bar graphs of moment burst size b_m (B) and moment burst frequency f_m (C) calculated from basal and TNF-stimulated smFISH distributions presented in Figs 1 and 2 for the indicated genes at the indicated time points. Error bars indicate bootstrapped 95% CIs. Significant differences indicated by non-overlapping CIs. Sample sizes are displayed in Figs 1E and 2C.

© 2021 The Authors

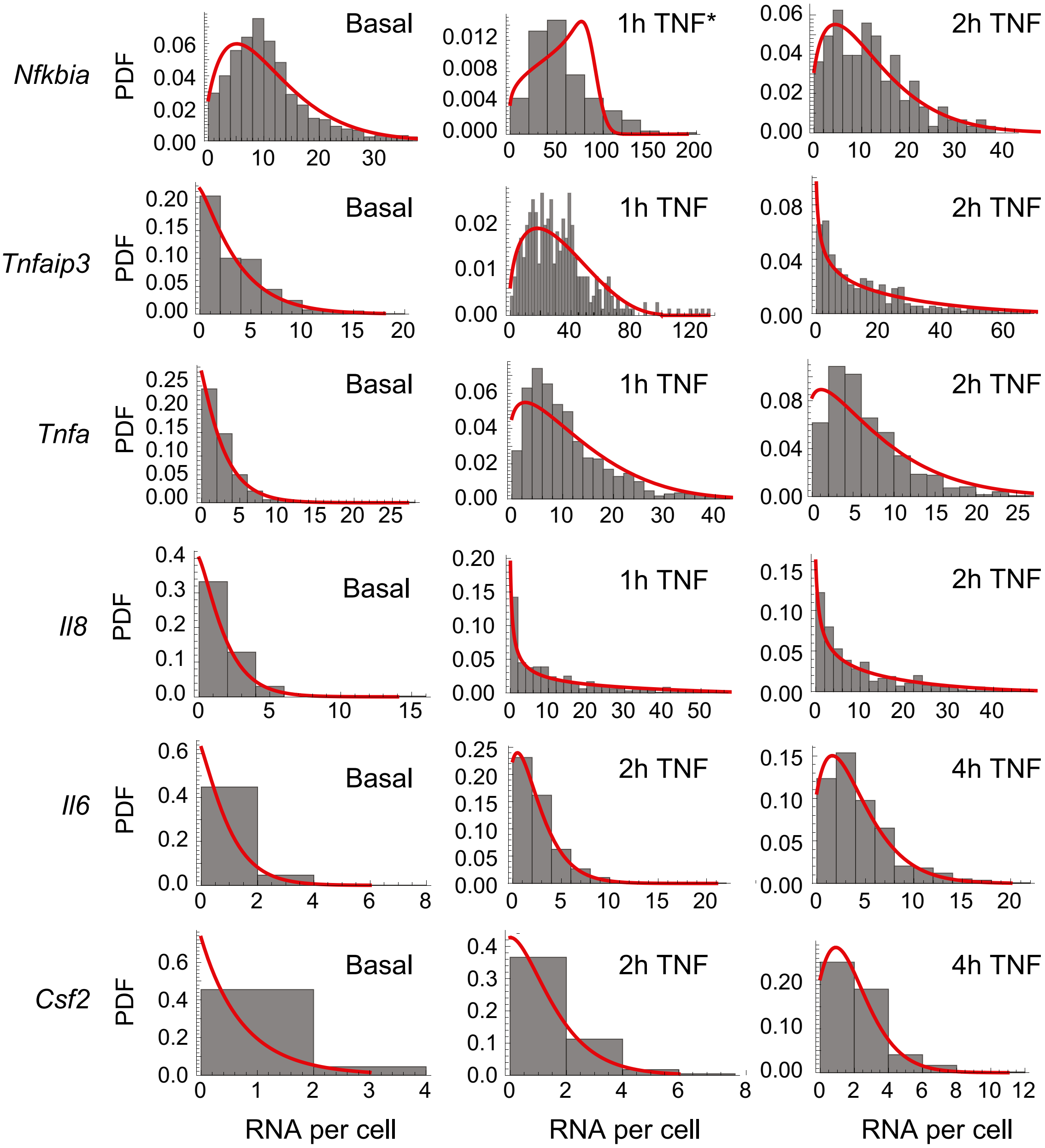

Figure EV3. smFISH distributions are well fit by the probability density function for the two-state promoter model (related to Fig 3).

Histograms (gray) of mRNA distributions for the indicated genes measured by smFISH in the basal state and after 20 ng/ml TNF treatment for combined experimental datasets unless indicated. Red curves show the best fit of the theoretical probability density function solution for the random telegraph model obtained by maximum likelihood estimation. *Fit for *Nfkbia* 1-h TNF distribution is for one replicate only (see Materials and Methods).

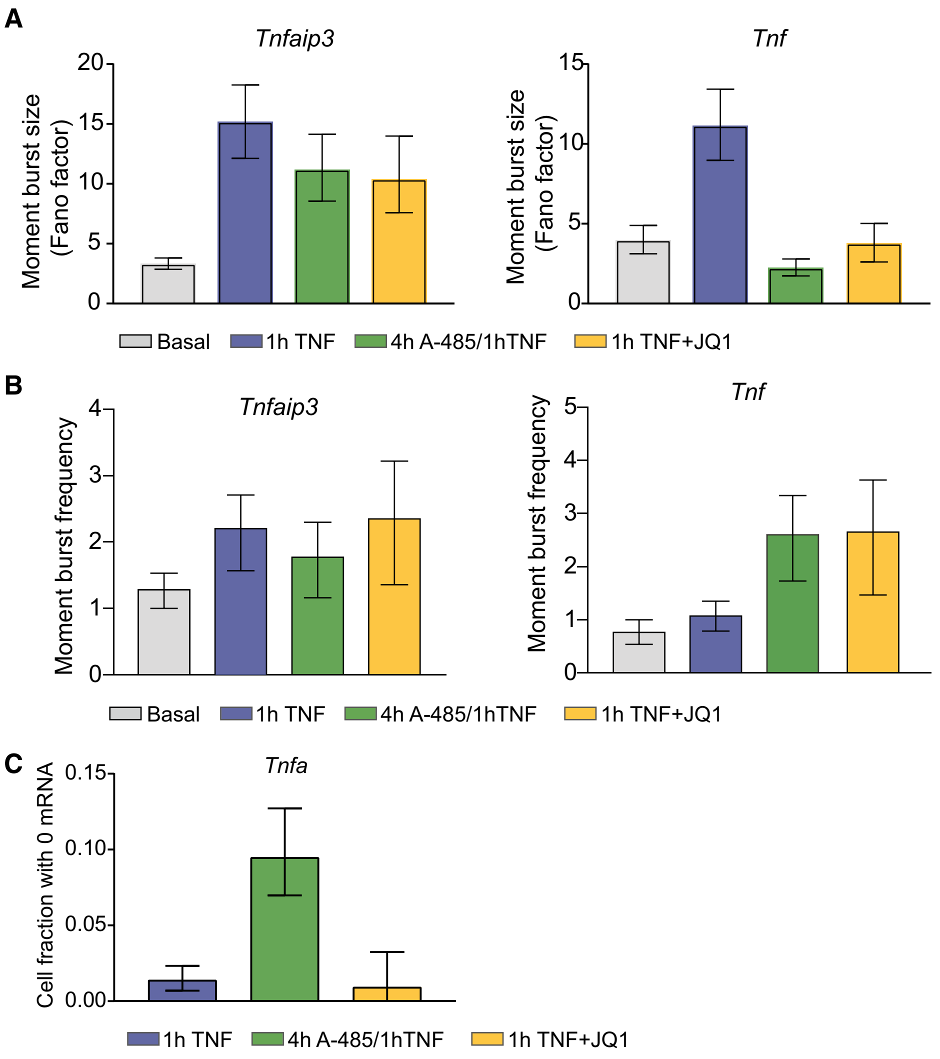

Figure EV4. A-485 and JQ1 differentially affect the fraction of non-responding cells in response to TNF treatment (related to Fig 5).

A, B Bar graphs of the moment burst size b_m (i.e., Fano factor) (A) and moment burst frequency f_m ($\mu/(b_m - 1)$) of TNF-stimulated smFISH distributions for basal (gray) or 20 ng/ml TNF for 1 h alone (blue) or in combination with a 4 h A-485 pretreatment (green) or with 1-h JQ1 (yellow). Error bars indicate bootstrapped 95% CIs. Sample sizes are displayed in Fig 5G and H.

C Fraction of cells with no *Tnf* transcripts after 1 h of TNF stimulation in combination with the indicated inhibitors as described in (A, B). Error bars indicate bootstrapped 95% CIs.

 © 2021 The Authors

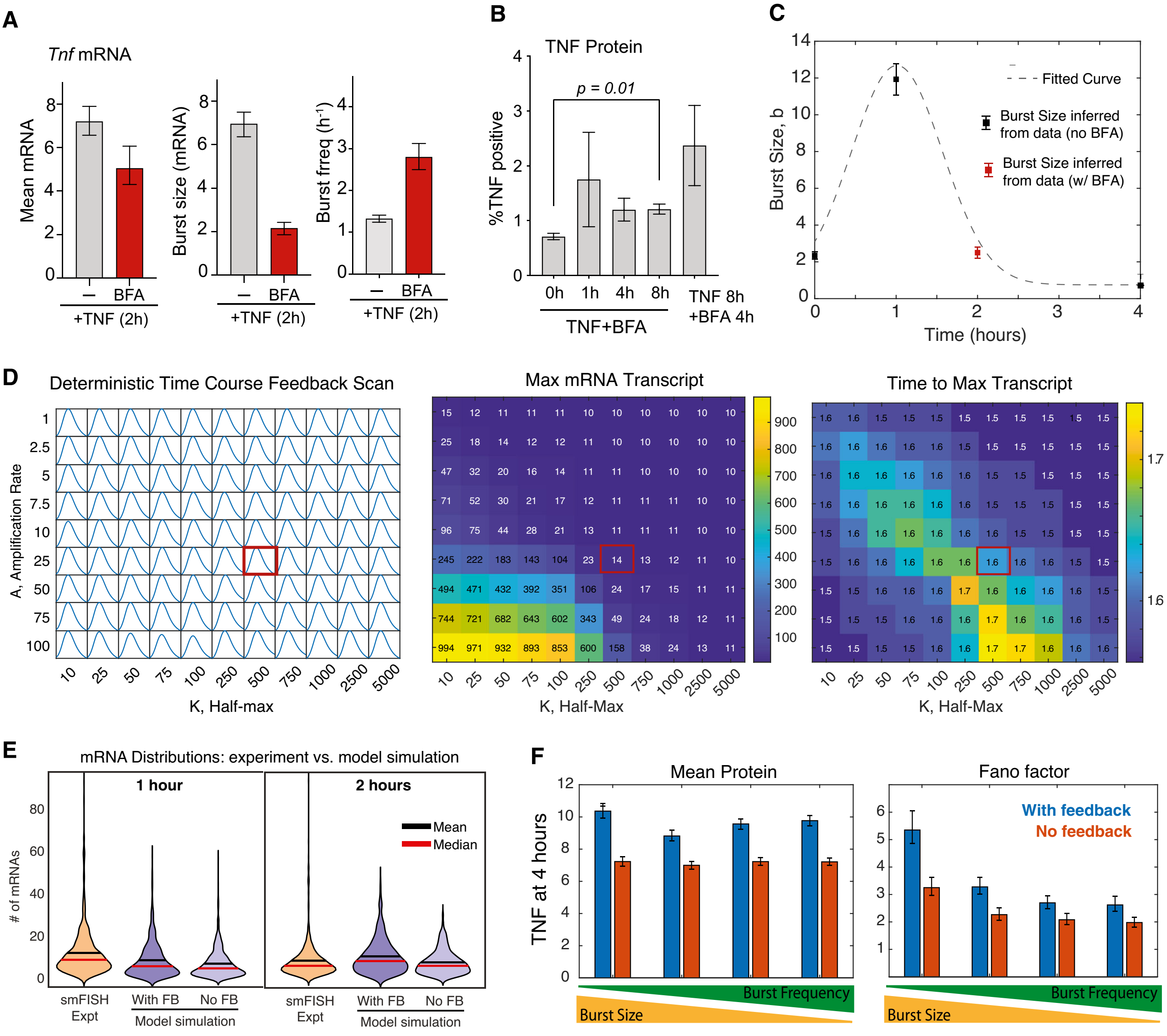

Figure EV5. Experimental data and model fitting for simulation of Tnf stochastic gene expression with positive feedback (related to Fig 6).

A Bar graphs of mean of *Tnf* smFISH distributions (left) and inferred burst size and burst frequency (right) after treatment with 20 ng/ml TNF for 2 h alone or in the presence of brefeldin A (BFA). Cells for TNF + BFA are from one replicate ($n = 148$). Error bars indicate bootstrapped 95% CIs.

B Bar graphs of % cells positive for intracellular TNF after treatment with 20 ng/ml TNF for 0, 1, 4, or 8 h in the presence of brefeldin A (BFA) or for 8 h TNF with BFA present at 4–8 h only. Data are presented as the mean ± s.e.m. of three biological replicates. Significance is reported if $P < 0.05$ by Welch's *t*-test.

C A time-dependent burst size function for *Tnf* mRNA production was fit to experimental data as indicated.

D Time course of *Tnf* mRNA (left), maximum mRNA (middle), and time to max mRNA (right) from a deterministic model simulation for varying amplification (a) and half-maximal value (*K*) to find feedback strength that approximately reproduces experimentally measured *Tnf* transcript levels (Fig 1B). The red box indicates the parameter set used to produce the graphs in Fig 6B and C.

E Violin plots of *Tnf* mRNA distributions measured by smFISH 1–2 h after TNF stimulation and simulated mRNA distributions with and without TNF positive feedback for the same conditions.

F Predicted mean and Fano factor for protein in simulated single-cell distributions 4 h after TNF treatment for four different bursting parameter sets. The "high burst size" parameters (left) and "high burst frequency" parameters (right) are calculated from the distributions presented in Fig 6D and E. Error bars represent 95% CIs from 1,000 single-cell simulations.

© 2021 The Authors
